# High Sucrose Diet-Induced Subunit I Tyrosine 304 Phosphorylation of Cytochrome *c* Oxidase Leads to Liver Mitochondrial Respiratory Dysfunction in the Cohen Diabetic Rat Model

**DOI:** 10.3390/antiox13010019

**Published:** 2023-12-21

**Authors:** Tasnim Arroum, Lucynda Pham, Taryn E. Raisanen, Paul T. Morse, Junmei Wan, Jamie Bell, Rachel Lax, Ann Saada, Maik Hüttemann, Sarah Weksler-Zangen

**Affiliations:** 1 Center for Molecular Medicine and Genetics, Wayne State University, Detroit, MI 48201, USA; ho0066@wayne.edu (T.A.); lucynda.pham@med.wayne.edu (L.P.); teraisanen20@my.trine.edu (T.E.R.); morsepa@wayne.edu (P.T.M.); am4472@wayne.edu (J.W.); jbell@dmc.org (J.B.); 2 Faculty of Medicine Hebrew, University of Jerusalem, Jerusalem 9112102, Israel; rachel.lax@mail.huji.ac.il (R.L.); ann.saadareisch@mail.huji.ac.il (A.S.); 3 The Hadassah Diabetes Center, Hadassah Medical Center, Jerusalem 9112102, Israel; 4 The Liver Research Laboratory, Hadassah Medical Center, Jerusalem 9112102, Israel; 5 Department of Genetics, Hadassah Medical Center, Jerusalem 9112102, Israel; 6 Department of Medical Laboratory Sciences, Hadassah Academic College, Jerusalem 9101001, Israel; 7 Department of Biochemistry, Microbiology, and Immunology, Wayne State University, Detroit, MI 48201, USA

**Keywords:** tyrosine phosphorylation, dimeric complex IV, inflammation, Cohen diabetic rat, cytochrome *c* oxidase, respiratory dysfunction, inhibitory phosphorylation, liver mitochondria, high sucrose diet, tyrosine 304 CcO subunit I, blue native gel, type 2 diabetes, mitochondrial dysfunction, mitochondria

## Abstract

The mitochondrial oxidative phosphorylation process generates most of the cellular energy and free radicals in mammalian tissues. Both factors play a critical role in numerous human diseases that could be affected by reversible phosphorylation events that regulate the function and activity of the oxidative phosphorylation complexes. In this study, we analyzed liver mitochondria of Cohen diabetes-sensitive (CDs) and Cohen diabetes-resistant (CDr) rats, using blue native gel electrophoresis (BN-PAGE) in combination with mitochondrial activity measurements and a site-specific tyrosine phosphorylation implicated in inflammation, a known driver of diabetes pathology. We uncovered the presence of a specific inhibitory phosphorylation on tyrosine 304 of catalytic subunit I of dimeric cytochrome *c* oxidase (CcO, complex IV). Driven by a high sucrose diet in both CDr and CDs rats, Y304 phosphorylation, which occurs close to the catalytic oxygen binding site, correlates with a decrease in CcO activity and respiratory dysfunction in rat liver tissue under hyperglycemic conditions. We propose that this phosphorylation, specifically seen in dimeric CcO and induced by high sucrose diet-mediated inflammatory signaling, triggers enzymatic activity decline of complex IV dimers and the assembly of supercomplexes in liver tissue as a molecular mechanism underlying a (pre-)diabetic phenotype.

## 1. Introduction

Mitochondrial dysfunction has long been suggested as a fundamental factor in the development of diabetic pathology across various organs [1,2]. However, the precise molecular mechanisms underlying this dysfunction have remained elusive. In this study, we employed the Cohen diabetic rat model, which was established through over 100 generations of selective inbreeding, resulting in distinct metabolically characterized rat strains: the Cohen diabetes-sensitive (CDs) and Cohen diabetes-resistant (CDr) rats [3]. This model proves valuable for investigating diet-induced type 2 diabetes (T2D) and understanding the influence of genetic susceptibility [4,5]. While CDr rats do not develop a T2D phenotype, CDs rats develop diabetes when exposed to a diabetogenic high sucrose-low copper diet (HSD), while maintaining normal blood glucose levels on a regular diet (RD). Hyperglycemia in CDs rats is characterized by elevated blood glucose levels, diminished insulin secretion in response to glucose loading, along with the presence of fatty liver, exocrine lesions, and infiltration of macrophages expressing the pro-inflammatory cytokine interleukin 1-β in the pancreas [4,6,7]. The proposed detrimental mechanism involving these macrophages includes increased nitric-oxide production and inhibited activity of cytochrome *c* oxidase (CcO, complex IV) [8]. This detrimental process leads to reduced ATP production, impaired insulin secretion, and the development of diabetes in CDs rats [3].

Our previous studies on pancreatic islets revealed mitochondrial dysfunction of the electron transport chain complex IV (cytochrome *c* oxidase, CcO) [6,8], the terminal enzyme of the electron transport chain, which catalyzes the proposed rate-limiting step of aerobic energy metabolism under physiological conditions [9,10,11,12]. CcO is highly controlled through the three known major regulatory mechanisms, i.e., allosteric regulation through the ATP/ADP ratio and thyroid hormones [13,14], expression of tissue specific isoforms [15], and posttranslational modifications, in particular phosphorylation [16]. Of the known phosphorylation sites on CcO, one is of particular interest: phosphorylation of CcO subunit I tyrosine 304 (Y304). Y304 is located near the catalytic pocket that binds oxygen [17] and can be phosphorylated through a cAMP-dependent mechanism [18] or inflammatory signaling [19] by a yet-to-be-identified downstream mitochondrial tyrosine kinase. Importantly, this phosphorylation leads to CcO inhibition [18,19].

Due to the link between heightened inflammatory signaling in diabetes impacting various organs [17,18,19] and considering the pivotal role of the liver in governing both lipid and glucose metabolism, we here investigated the importance of mitochondrial dysfunction in the liver using the Cohen rat model. Mitochondrial dysfunction may play a more central role in the pathology of diabetes than originally anticipated. We were also specifically interested in analyzing CcO monomers and dimers, in addition to the higher-order assemblies of the electron transport complexes, known as supercomplexes (SCs). To address this, we employed Blue Native Polyacrylamide Gel Electrophoresis (BN-PAGE) analysis, a powerful technique for examining the higher-order protein assemblies of the electron transport chain complexes and their activity within a native gel [20,21]. In the BN-PAGE gel, proteins retain their native conformation and exist in different multiprotein complex stoichiometries [22]. These SCs are substantial multi-protein structures primarily composed of complex I (NADH:ubiquinone oxidoreductase, CI), complex III (ubiquinol:cytochrome *c* oxidoreductase, CIII), and complex IV (cytochrome *c* oxidase, CcO, CIV). Diverse supercomplexes have been observed in various organisms, including fungi, mammals, plants, and algae [23]. They are formed by different combinations of complexes I, III, and IV. For instance, (i) the “I+III_2_ supercomplex” consists of complex I and dimeric complex III; (ii) the “III_2_+IV_1–2_ supercomplexes” are composed of dimeric complex III and one to two copies of monomeric complex IV; and (iii) the “I+III_2_+IV_1–4_ supercomplexes” are extensive structures composed of complexes I, dimeric complex III, and one to four copies of complex IV [23]. Their formation and regulation are not well understood but they have been proposed to facilitate ETC flux. Interestingly, after mechanistically examining the electron transport chain in liver tissue structurally and functionally we observed a reduction in liver CcO activity in both CDs and CDr animals following HSD. At the molecular level, there was an increase in CcO Y304 phosphorylation. This phosphorylation facilitates the formation of CcO dimers and subsequent CcO inhibition, pointing to a new molecular metabolic pathway implicated in T2D.

## 2. Materials and Methods

### 2.1. Cohen Diabetes-Sensitive and Cohen Diabetes-Resistant Rats Tissue Harvesting

**Figure 1:**
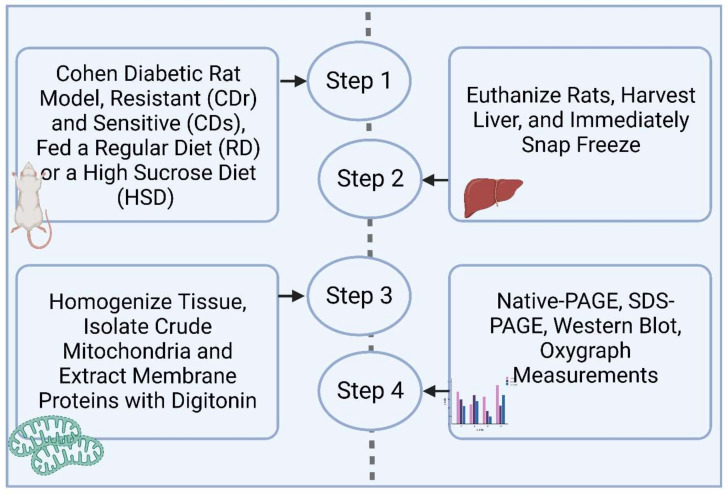
Schematic representation of the applied workflow to study electron transport chain composition and activity in the Cohen diabetes rat model.

The Cohen rat diabetes animal model is an experimental model of nutritionally induced diabetes developed by selective inbreeding [3,4,6]. The Cohen rats have a genetic predisposition to develop hyperglycemia along with fatty pancreas and liver [7]. Male Cohen diabetes-sensitive (CDs) and Cohen diabetes-resistant (CDr) rats were fed a high sucrose diet (HSD) or regular diet (RD) for 1 month. HSD diet composition was used as described in detail [6]. CDs and CDr rats were bred and maintained in the animal facility at the Hebrew University School of Medicine in Jerusalem. The rats were initially fed a regular rodent diet (Teklad, 2018, Harlan Laboratories, Placentia, CA, USA) as described [5]. At six weeks of age, male rats on RD were transitioned to HSD, specifically a custom-prepared diabetogenic high-sucrose, low-copper diet and is comprised of 72% sucrose, 18% vitamin-free casein, 5% salt mixture no. II USP (MP Biomedicals, Solon, OH, USA), 4.5% butter, 0.5% corn oil, vitamins, and low copper (1.2 ppm) [5]. The study included four groups: two groups were fed an RD (CDs RD and CDr RD) and two groups fed an HSD (CDs HSD and CDr HSD). Glucose concentrations were measured in tail blood samples using a standard glucometer (Elite, Bayer, Leverkusen, Germany). Blood glucose levels were assessed after an overnight fast, following the oral administration of 3.5 g/kg glucose [5]. Hyperglycemia was defined as blood glucose levels > 11 mmol/L two hours post-oral glucose tolerance test (OGTT), while normoglycemia was defined as <7.8 mmol/L. Rats were euthanized using an overdose of a combined ketamine/xylazine mixture and livers were immediately removed and flash-frozen in liquid nitrogen and stored at −80 °C until processing. Animal experiments were approved under protocol number MD-22-16924-3 by the Institutional Animal Experiments Committee (IACUC). Subsequently, liver mitochondria were isolated and analyzed. Figure 1 provides an overview of the experimental process. As shown in Table 1, only CDs HSD rats presented with a diabetic phenotype via OGTT. Data are means ± SEM * *p* < 0.01 relative to CDr RD.

### 2.2. Liver Tissue Homogenization and Mitochondrial Enrichment for Blue Native PAGE (BN-PAGE)

Liver tissue (30 mg) was homogenized using a glass-Teflon homogenizer (Grainger, Lake Forest, IL, USA) with 5 strokes at 1500 rpm in 2 mL of mitochondrial isolation buffer (MIB: 200 mM sucrose, 10 mM Tris, 1 mM EGTA, pH 7.4 adjusted with 1 M HEPES solution, Gibco, cat. No. 15630080). To preserve protein integrity, we added 1 mM phenylmethylsulphonyl fluoride and 1 mM heat-activated sodium vanadate to the homogenization buffer and the entire process was conducted on ice. To enrich the mitochondrial fraction, we employed a series of centrifugation steps. First, a low-speed centrifugation (800× *g*, 10 min, 4 °C) was used to remove large cellular debris and nuclei, leaving the supernatant containing mitochondria and other organelles. The supernatant was then subjected to another round of centrifugation under the same conditions to further reduce contaminants. Finally, the supernatant was centrifuged at higher speed (17,000× *g*, 10 min, 4 °C) to precipitate the crude mitochondrial fraction.

### 2.3. Mitochondrial Membrane Solubilization for BN-PAGE

The BN-PAGE protocol was adapted from [21,24]. All components for the subsequent steps were used from the NativePAGE™ Sample Prep Kit (ThermoFisher Scientific, Waltham, MA USA, cat. no. BN2008) including NativePAGE™ 4× sample buffer, NativePAGE™ 5% G-250 sample additive, and digitonin (5%). To release mitochondrial proteins embedded within the inner mitochondrial membrane while preserving their higher molecular structures under non-denaturing conditions, we employed the gentle detergent digitonin. However, as digitonin can precipitate, it was essential to heat the solution at 95 °C for 5 min, gently vortex to dissolve any precipitate, and then cool it on ice before use. For solubilizing crude mitochondria, we used NativePAGE™ 4× sample buffer supplemented with digitonin (5%) to reach a final concentration of 2% (digitonin/protein ratio of 8 g/g). This mixture was then incubated on ice for 20 min to extract the protein complexes from the inner mitochondrial membrane. Subsequently, we carried out a centrifugation step at 4 °C and 17,000× *g* for 30 min. Following centrifugation, the supernatant containing the unbound proteins was supplemented with 0.5% G-250 sample additive. Protein concentration was determined using the DC protein assay kit (Bio-Rad, Hercules, CA, USA, cat. no. 5000111) according to the manufacturer’s protocol. Subsequently, 50 µg of these samples were loaded onto 3–12% native gradient gels (Invitrogen, Carlsbad, CA, USA, cat. no. BN1001BOX). Prior to use, both the blue cathode buffer (50 mM Tricine, 15 mM Bis-Tris, 0.02% G-250 dye, pH 7.0) and the anode buffer (50 mM Bis-Tris, pH 7.0) were prechilled. Electrophoresis was conducted at 4 °C, applying 100 V until the proteins entered 1/3 into the gel when the voltage was increased to 150 V, until the desired separation was achieved.

### 2.4. Mitochondrial Native Protein In-Gel Activity Assay (IGA)

After the BN-PAGE, we conducted an in-gel activity assay (IGA) as described in [21]. The gel was incubated in 20 mL freshly made complex assay buffer at room temperature. CcO assay was conducted first, then in the same gel, complex I (CI) assay was performed: The CcO activity buffer contains: 50 mM phosphate buffer pH 7.4, 0.5 mg/mL 3,3′diaminobezidine tetrachloride (DAB: Sigma, Burlington, MA, USA, cat. no. D5637), 1 mg/mL cytochrome *c* (Sigma, cat no. C3131). The CI activity buffer contains: 2 mM Tris-Cl, pH 7.4, 0.1 mg/mL NADH (Sigma, cat. no. N4505), 2.5 mg/mL nitrotetrazolium blue chloride (NTB: ThermoFisher, cat. no. J60230.03). Both reactions were carried out at 20 °C for 6 h and images were recorded every hour. We used the pictures obtained after 1 h of incubation for quantitation when the reactions/signals were not saturated.

### 2.5. Cytochrome c Oxidase Activity Measurements Using the Polarographic Method

To examine the effect of a HSD on respiration in the Cohen CDr and CDs rat strains, we measured CcO activity using a Clark-type oxygen electrode (Oxygraph system, Hansatech, Norfolk, UK) at 25 °C. Mitochondrial fractions were enriched from the liver, as described above. Protein concentration was determined using the DC protein assay kit (Bio-Rad). For each measurement, 30 µg of total mitochondrial protein was used. The mitochondrial pellet was resuspended in a solubilization buffer (10 mM K-HEPES, pH 7.4; 40 mM KCl; 1% Tween 20; 1 mM phenylmethylsulphonyl fluoride; 1 mM heat-activated sodium vanadate; 10 mM KF; 2 mM EGTA). Subsequently, mitochondrial membranes were disrupted using a Fisher Scientific (Waltham, MA, USA) Sonic Dismembrator Model 100 ultrasonicator (three rounds of 5-s pulses). Oxygen consumption was assessed in the presence of 20 mM ascorbate and 30 µM cytochrome *c* (Sigma, cat no. C3131). Oxygen consumption rates were continuously monitored, and the data were recorded in nmol/min/mg mitochondrial protein and analyzed with the OxyTrace+ software (version 1.0 Build 48, 2016, Hansatech Instruments Ltd., Amesbury, MA, USA).

### 2.6. Western Blot Analysis

After solubilizing the mitochondrial membranes for BN-PAGE, mitochondrial proteins were either directly transferred from the native gel to a PVDF membrane for studying native protein complex structures, or the remaining isolated mitochondria (20 µg) were taken from samples and mixed with RIPA buffer (50 mM Tris-HCl, pH 8.0, 150 mM NaCl, 1% Nonidet P 40, 0.5% sodium deoxycholate, 0.1% SDS, supplemented with protease and phosphatase inhibitor cocktails PhosSTOP Phosphatase, Complete Protease Inhibitor Cocktail, Roche Applied Science, Indianapolis, IN, USA). Loading buffer (4% SDS, 10% 2-mercaptoethanol, 20% glycerol, 0.004% bromophenol blue, 0.125 M Tris-HCl, adjusted pH to 6.8) was added to the samples and incubated for 20 min at 45 °C. Next, we loaded the samples onto a 10% tris-tricine SDS-PAGE gel operated at 100 V followed by an overnight wet transfer at 25 V or 140 mA for 1h at 4 °C. The transfer buffer (25 mM Tris base 192 mM glycine, 20% methanol, 0.1% SDS, pH adjusted to 8.3) contained SDS to facilitate the transfer of large protein complexes. Membranes were blocked with a blocking reagent (5% bovine serum albumin (BSA), 1× Tris-buffered saline with Tween-20: TBST, 0.1% Tween-20) for 1 h at room temperature. It is crucial to not use dried milk as blocking reagent when studying phosphorylated proteins. In addition, when studying the CcO subunit I, heating of mitochondrial proteins beyond 45 °C should be avoided, as higher temperatures may lead to subunit I aggregation which can disrupt antibody–protein epitope binding. Next, we applied the primary antibody (Table 2) for an overnight incubation at 4 °C with gentle shaking or for 90 min at room temperature. In cases where the membranes were stained with Coomassie, a 5 min methanol wash step was performed to remove the dye to improve antibody binding. Subsequently, the membranes were washed three times, each for 10 min, using TBST as the wash solution. Finally, membranes were incubated with the secondary antibody for 1 h at room temperature (Table 2), then overlaid with Pierce™ ECL Western Blotting Substrate (ThermoFisher, cat. no. 32106), and signals were recorded on an X-ray film. Production of our anti-pY304 CcO subunit I specific antibody is described in detail [19]. Briefly, customized polyclonal antibodies were generated against the phosphotyrosine 304 epitope of CcO subunit I by Abgent (San Diego, CA, USA) after immunizing rabbits with the cysteine-conjugated synthetic peptide GMDVDTRApYFTSAC. Antibodies detecting the unphosphorylated sequence were removed by affinity absorption against column-bound cysteine-conjugated synthetic GMDVDTRAYFTSAC peptide.

### 2.7. ATP Measurements

ATP levels were assessed using the boiling method on flash-frozen liver tissue samples, utilizing the ATP bioluminescence assay kit HS II from Roche Applied Science (catalog # 11 699 709 001). The ATP release procedure was initiated with the addition of 600 µL of boiling buffer (100 mM Tris-Cl, pH 7.75, 4 mM EDTA) to the samples, promptly followed by their transfer to a boiling water bath for 2 min. Then, samples were cooled on ice and subjected to sonication. Subsequently, measurements were executed using the ATP bioluminescence assay kit HS II following the manufacturer’s specifications. ATP concentrations were normalized to beta-actin levels in tissue through SDS-PAGE followed by Western blot analysis.

### 2.8. Statistical Analyses

All data are presented as mean ± SEM (standard error of the mean) values. To assess the differences between HSD and RD in both rat strains, i.e., Cohen diabetes-sensitive (CDs) and Cohen diabetes-resistant (CDr) rats, we conducted a two-way ANOVA followed by a post hoc Bonferroni test. These statistical analyses were carried out using Statistical software from OriginLab Corporation (Version 2023b, Northampton, MA, USA) with a significance level set at *p* ≤ 0.05.

## 3. Results

### 3.1. CcO Activity Is Decreased in CDr and CDs Rat Liver Mitochondria following HSD

In previous studies, alterations in homogenates of islets and peripheral-blood mononuclear cells (PBMCs) from CDr and CDs strains were observed. CcO activity gradually decreased associated with the period of time the animals were exposed to the diabetogenic diet [6]. However, the HSD impacts liver mitochondrial function and fatty infiltration, and the mechanism underlying these observations has not yet been conducted.

**Figure 2:**
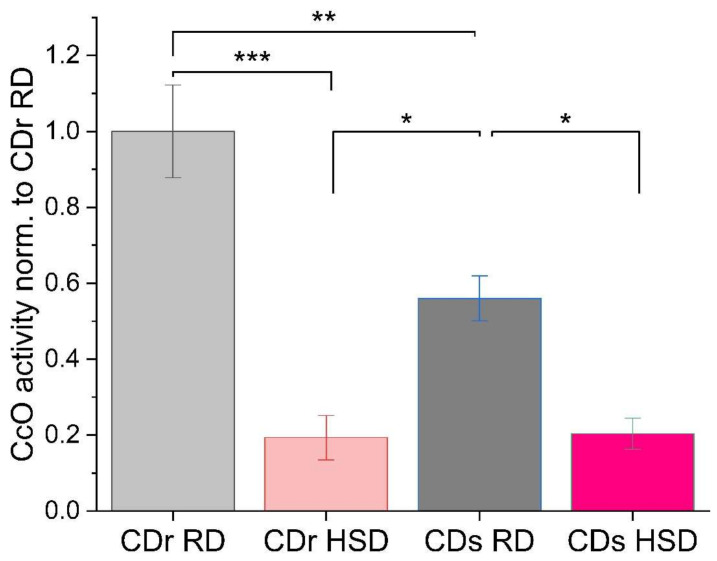
High sucrose diet leads to a reduction in enzymatic activity of cytochrome *c* oxidase (CcO) in both Cohen diabetes-resistant (CDr) and Cohen diabetes-sensitive (CDs) rats. CcO specific activity of sonicated rat liver mitochondria was analyzed with a Clark-type electrode in the presence of 30 µM cytochrome *c*. RD, regular diet; HSD, high sucrose low copper diet. Data were collected from 4 independent experiments using mitochondria from four different rat livers per group and normalized to total mitochondrial protein; data are reported as % of CDr RD set to 100% (CDr RD OCR 100% is equivalent to 64.7 nmol O_2_ consumption/min/mg mitochondrial protein; ***, *p* < 0.001; **, *p* < 0.01; *, *p* < 0.05.

First, oxygen consumption rate (OCR) of cytochrome *c* oxidase (CcO or complex IV) was analyzed using an oxygen electrode (Oxygraph). CcO is the proposed rate-limiting enzyme in the electron transport chain under normal physiological conditions [25], and its regulation plays a crucial role in mitochondrial function. Two-way ANOVA analysis revealed significant main effects associated with both factors, strain and diet composition. Notably, strain type exerted a statistically significant influence on OCR, as confirmed by the *F* statistic (*F*(strain) = 7.844, *p* < 0.05). Simultaneously, a profound main effect of diet composition on OCR was unveiled, with a robust *F* value (*F*(diet) = 57.546, *p* < 0.0001). This outcome highlights the influence of dietary composition, whether characterized by a regular or high sucrose diet, on the overarching profile of oxygen consumption. Moreover, the interaction between strain and diet emerged as a significant player (*F* = 8.62178, *p* < 0.05). This implies that the combined influence of strain type and diet is more than the sum of their individual effects. The comparison between the individual groups is presented in Figure 2, with a profound decrease in CcO enzymatic activity seen in both strains when they were subjected to the HSD. The CDr strain exhibited an 80% reduction, while the CDs strain experienced a 36% reduction in CcO activity. Interestingly, on the regular diet, when the CDs rats are still normoglycemic, the CDs rats already displayed a 44% reduction compared to the CDr RD group. This observation underscores the vulnerability and heightened susceptibility of the CDs strain to mitochondrial dysfunction, emphasizing the importance of diet and strain-related factors in the development of a (pre-)diabetic phenotype.

### 3.2. Analysis of Electron Transport Chain Supercomplex Composition and Activity Reveal Pronounced Reduction of CcO Activity in the Dimeric Enzyme

**Figure 3:**
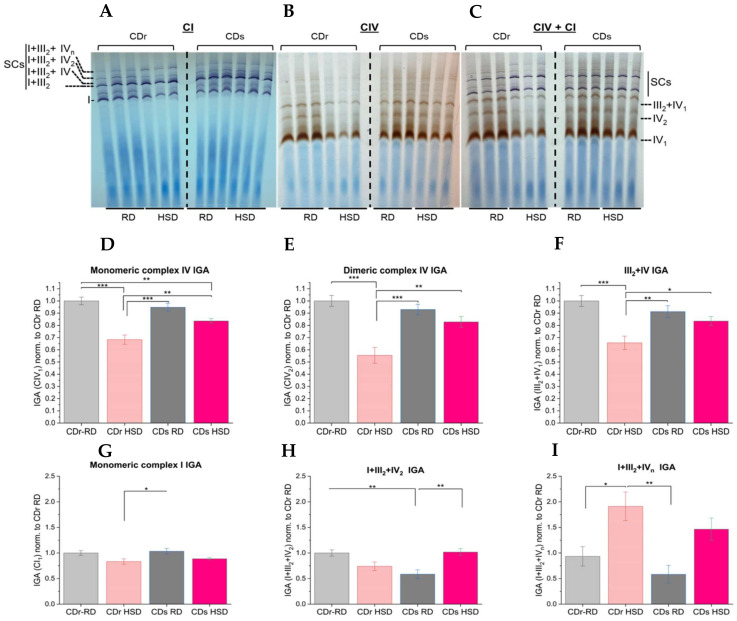
High resolution in-gel activity assessments reveal reduced activity of dimeric complex IV. Annotation of supercomplexes and native protein complexes are provided in the figures. (**A**) Gels were incubated with complex I substrate and violet bands represent complex I in-gel activity. (**B**) Gels were incubated with CcO substrate and brown bands indicate CcO (complex IV, CIV) in-gel activity. (**C**) Combined activities of complex I and complex IV. Quantification of monomeric (**D**) and dimeric CcO activity (**E**). (**F**) Quantitation of CcO activity as part of the III_2_-IV supercomplex. Quantification of monomeric complex I activity (**G**), complex I activity in the supercomplex I+III_2_+IV_2_ (**H**), and complex I activity in the supercomplexes I+III_2_+IV_n_ (**I**). SC denotes supercomplex. SC I+III_2_ comprises CI and a dimer of CIII. SC I+III_2_+IV consists of CI, a dimer of CIII, and monomeric CIV. SC I+III_2_+IV_2_ includes CI, a dimer of CIII, and a dimer of CIV. SC I+III_2_+IV_n_ is composed of CI, a dimer of CIII, and a variable (n) number of CIV. SC III_2_+IV_1_ consists of dimeric CIII and monomeric CIV. IV_2_ and IV_1_ represent dimeric and monomeric CIV, respectively. I denotes monomeric CI. Data derived from three independent experiments using mitochondria from three distinct rat livers per group ***, *p* < 0.001; **, *p* < 0.01; *, *p* < 0.05.

While the oxygen electrode provides precise OCR measurements, it does not reveal variations in the activities of different proteins within the higher-order assemblies of the electron transport complexes, ranging from CcO monomers and dimers to SCs. We were particularly interested in characterizing respiratory supercomplexes within distinct CDr and CDs strains under the two dietary conditions using BN-PAGE. To maintain the integrity of the supercomplex structures and protein complexes in their native state, we used digitonin as a mild detergent to solubilize mitochondrial membranes, maintaining the SC structures and protein complexes in their native state [24]. Following the separation of protein complexes in native PAGE, in-gel activity assays (IGA) were conducted to analyze enzyme activity across different complexes within the same sample and among the different groups (Figure 3). When comparing both the HSD and RD groups, in agreement with the measurements from the oxygen electrode, a general decrease in CcO activity was observed in the HSD groups. Notably, the most significant reduction was observed for dimeric CcO (IV_2_) of the CDr HSD group exhibiting a 45% reduction in activity compared to the CDr RD group (Figure 3, middle and right gels). In comparison, the CDs HSD group showed a lower, 11% reduction in dimeric CcO activity when subjected to HSD (Figure 3E). Complex IV-related in-gel activity is quantified in Figure 3D–F. Two-way ANOVA revealed that the composition of the diet emerged as a pivotal determinant significantly influencing the activity of all complex IV-containing complexes, encompassing monomer, dimer, and III_2_+IV configurations whereas the main effect of strain type was statistically non-significant. Of particular interest, we found a significant interaction effect between strain type and diet, highlighting the importance in considering both factors, to comprehensively decipher the observed changes in complex IV related activity.

**Figure 4:**
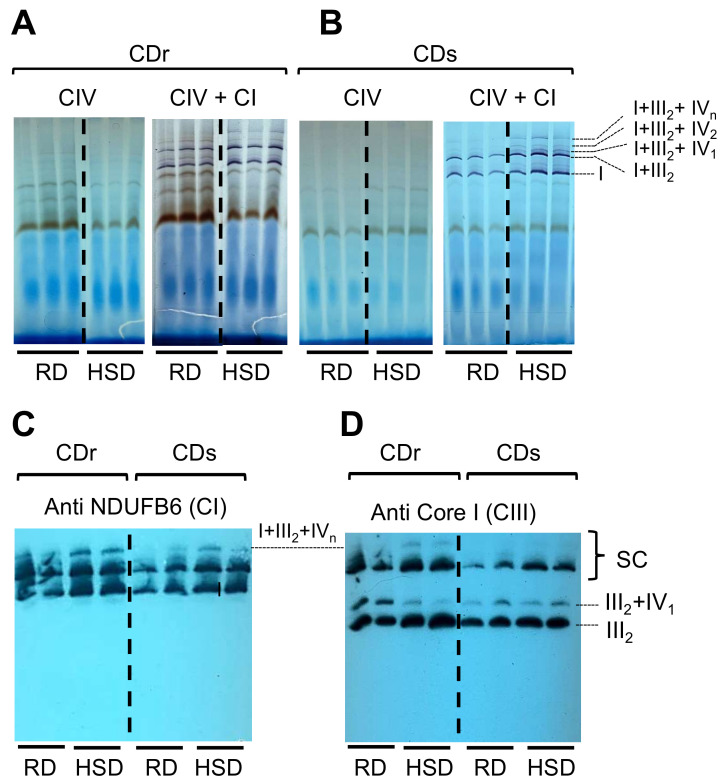
Shift of complex I in-gel activity towards higher supercomplexes following HSD. Top panels present aligned gels displaying in-gel activity assays of complex I and IV for (**A**) CDr and (**B**) CDs strains following BN-PAGE. (**C**) Western blot analysis following BN-PAGE with anti-NDUFB6 (CI) (**C**) and anti-Core I (CIII) antibodies (**D**) for the CDr and CDs strains as indicated. SC denotes supercomplex. SC I+III_2_ comprises CI, a dimer of CIII. SC I+III_2_+IV consists of CI, a dimer of CIII, and monomeric CIV. SC I+III_2_+IV_2_ includes CI, a dimer of CIII, and a dimer of CIV. SC I+III_2_+IV_n_ contains CI, a dimer of CIII, and a variable (n) number of CIV. SC III_2_+IV_1_ consists of dimeric CIII and monomeric CIV. III_2_ represents dimeric CIII. I denotes monomeric CI.

Complex I-related IGA analysis indicated no significant main effect for both strain and diet. Interestingly, however, the interaction effect of both factors played a significant role in driving changes in complex I-related IGA emphasizing the nuanced contributions of both elements to the observed alterations in Complex I related activity (the summary of the different *F* and *p* values of the IGA analysis are reported in Supplementary Table S1). There also was a redistribution of complex I activity, where the higher SC structures appear more prominent in the HSD groups in comparison to the RD groups. The quantifications of in-gel activity assay of complex I revealed no change in monomeric complex I (Figure 3G) and no change in I+III_2_ activity (Supplemental Figure S1). Interestingly, increased complex I activity shifted towards higher SCs composed of I+III_2_+IV_2–4_ following HSD both in the CDr and CDs groups (Figure 4A,B). When analyzing complex I with an NDUFB6 antibody following 1-D BN-PAGE, we found an increase in the amount of complex I protein at the higher respirasome multi-protein complexes (Figure 4C, I+III_2_+IV_n_), which correlated with an increase in activity of complex I related SCs. Finally, immunoblotting with a complex III (Core subunit I, Core I) antibody showed a decrease in complex III within the III_2_+IV band (Figure 4D).

### 3.3. High-Sucrose Diet Leads to Phosphorylation of CcO Subunit I Tyrosine 304 in the CcO Dimer

**Figure 5:**
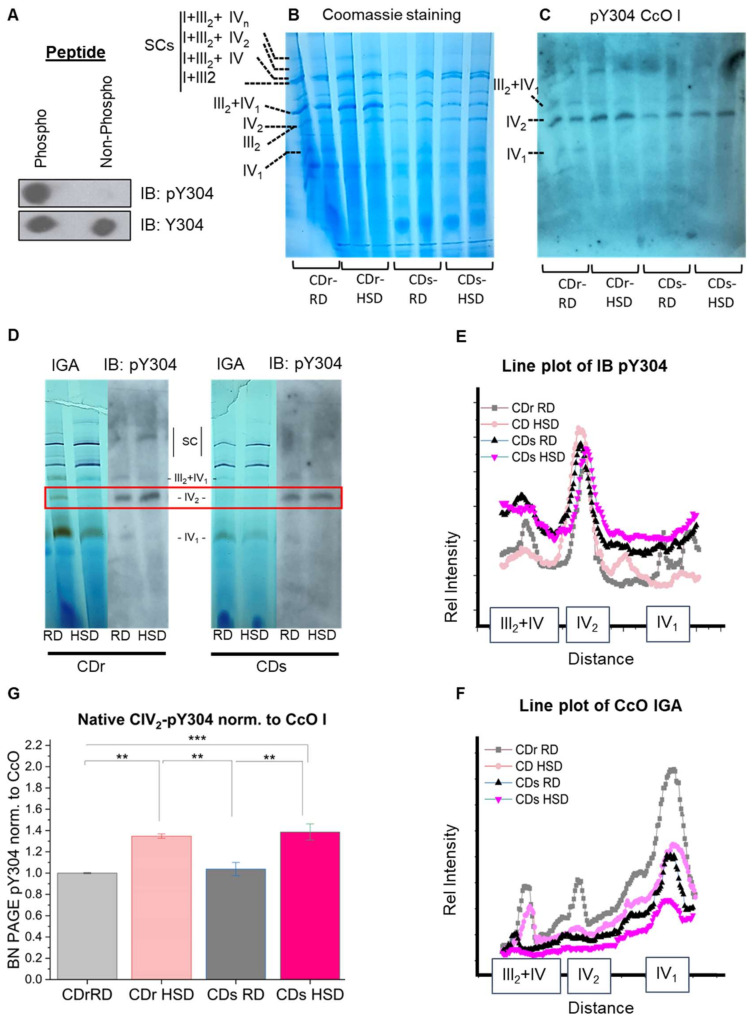
Diet-Induced CcO subunit I tyrosine 304 phosphorylation (pY304) is specific to the CcO dimers. (**A**) Dot blots of synthetic phosphorylated peptide (pY304: GMDVDTRApYFTSAC) and a synthetic non-phospho-specific peptide (Y304: GMDVDTRAYFTSAC) demonstrates specificity for the Y304-phosphorylated peptide only. The custom-made anti-pY304 CcO subunit I antibody was characterized in [19]. (**B**) Coomassie stained BN-PAGE and (**C**) BN-PAGE followed by Western blot analysis with a pY304-specific antibody, showing two representative experiments. (**D**) Gel alignment of BN-PAGE Western blot analysis with pY304 and in-gel activity assay of complex I (violet bands) and complex IV (brown bands), showing a representative experiment. (**E**) Representative line plot analysis of pY304 phosphorylation signal following BN-PAGE shown in (**C**). (**F**) Representative line plot analysis of complex IV in-gel assay signal in BN-PAGE shown in (**D**). (**G**) Quantification of relative phosphorylation of dimeric complex IV (CIV_2_) from the BN-PAGE normalized to CcO subunit I. SC denotes supercomplex. SC I+III_2_ comprises CI and a dimer of CIII. SC I+III_2_+IV consists of CI, a dimer of CIII, and monomeric CIV. SC I+III_2_+IV_2_ includes CI, a dimer of CIII, and a dimer of CIV. SC I+III_2_+IV_n_ encompasses CI, a dimer of CIII, and a variable (n) number of CIV. SC III_2_+IV_1_ consists of dimeric CIII and monomeric CIV. IV_2_ and IV_1_ represent dimeric and monomeric CIV. III_2_ is dimeric CIII. I is monomeric CI. Data were collected from four different experiments, ***, *p* < 0.001; **, *p* < 0.01.

We and others have shown that posttranslational modifications (PTMs) and in particular phosphorylations of the electron transport chain complexes play a pivotal role in regulating enzyme activity (reviewed in [16]). These modifications serve as a feedback mechanism to adapt cellular respiration to changing pathophysiological conditions or in response to stressors. In a previous study, we showed through mass spectrometry that tyrosine 304 (Y304) of CcO catalytic subunit I can be phosphorylated via a cAMP-dependent mechanism in liver and lung tissue [18]. Moreover, we later established that the same residue is targeted for phosphorylation by inflammatory signaling [19]. Y304 is located near the catalytic oxygen binding site of subunit I [17], and acts as a switch of enzyme activity, resulting in CcO inhibition and pronounced sigmoidal kinetics when phosphorylated [17,19]. Building on the knowledge that downstream inflammatory signaling causes CcO phosphorylation [19], we tested the hypothesis that the diminished activity of liver CcO in the observed (pre-)diabetic metabolic phenotype may be the result of PTMs such as Y304 phosphorylation of CcO subunit I. Therefore, we conducted immunoblotting following 1D BN-PAGE using our custom-made anti-CcO subunit I-pY304 specific antibody that only recognizes the phosphorylated epitope (Figure 5A) [19]. Our results revealed a significant accumulation of pY304 in CcO subunit I (Figure 5C), specifically within the CcO dimers (IV_2_) (see Figure 5B for monomer and dimer positions). Furthermore, the CcO monomer predominantly contained subunit I that was not phosphorylated (Figure 5D–F; quantitated in Figure 5G).

**Figure 6:**
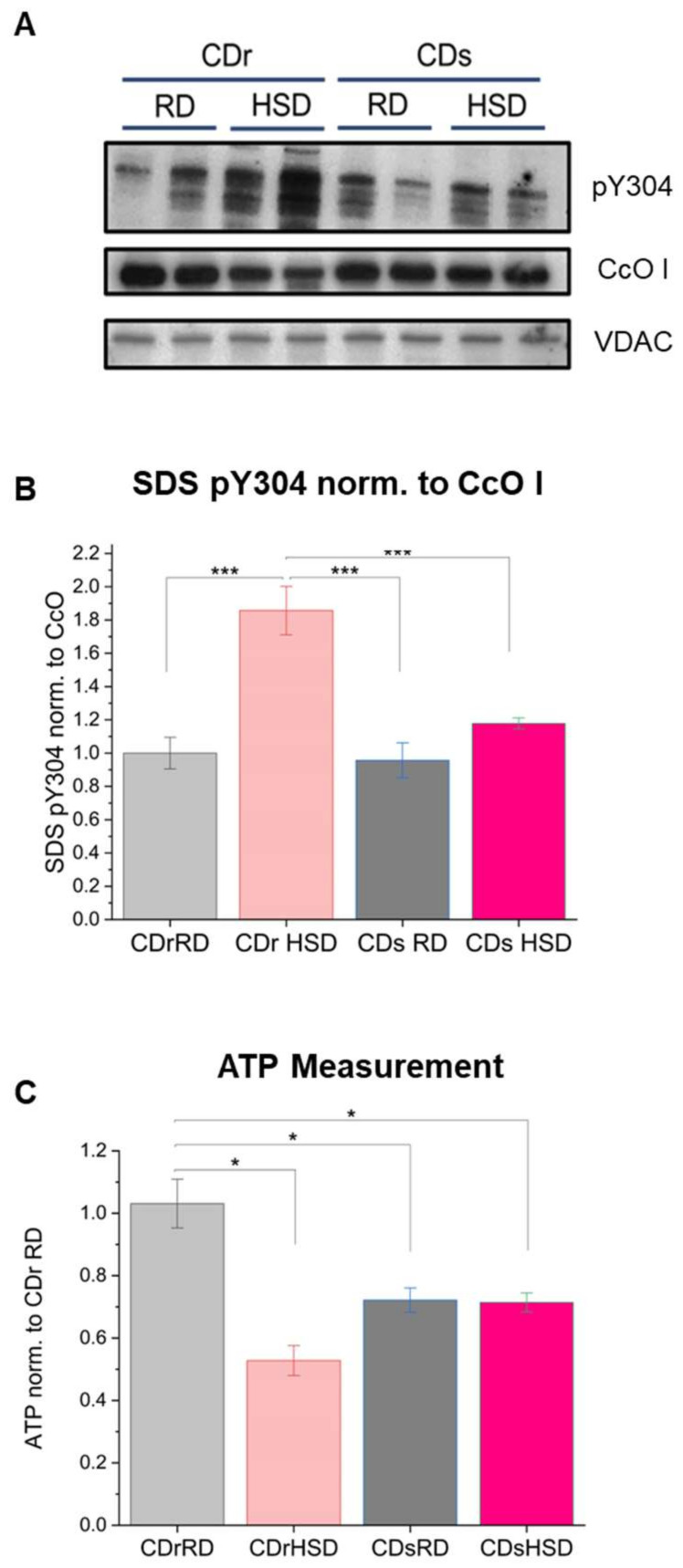
High sucrose diet leads to increased total Y304 phosphorylation of CcO subunit I and decreased ATP production in Cohen diabetes-resistant (CDr) rats. (**A**) Western blot analysis following SDS PAGE used for the normalization of pY304 BN PAGE analysis. (**B**) Quantitation of CcO subunit I Y304 phosphorylation normalized to total CcO subunit I following SDS PAGE. (**C**) ATP readings were normalized to ß-actin levels and reported as arbitrary units. RD, regular diet; HSD, high sucrose low copper diet. Data were collected from four different experiments, ***, *p* < 0.001; *, *p* < 0.05.

These findings suggest that the decrease in respiration can primarily be attributed to Y304 phosphorylation of CcO subunit I, which promotes complex IV dimer formation and inhibition of enzyme activity. To assess the extent of phosphorylation, we separated the mitochondrial proteins via SDS-PAGE and analyzed blots using our custom-made CcO subunit I pY304 antibody, a commercial CcO subunit I antibody (non-phospho specific), and an anti-VDAC antibody as control for mitochondrial mass. Figure 6A,B show a 22% increase in Y304 phosphorylation of the CcO subunit I in CDr HSD and a 12% increase in CDs HSD. Additionally, there was a 13% decrease in total CcO amount in the HSD liver mitochondria for both strains when fed a HSD (Supplemental Figure S2). These observations are consistent with our earlier findings, which indicated sigmoidal CcO kinetics for the Y304-phosphorylated enzyme, a characteristic of dimeric CcO that is inhibited by phosphorylation [18]. The two-way ANOVA analysis of CcO subunit I Y304 phosphorylation from SDS PAGE (Figure 6B) revealed a significant main effect of strain (*F* = 12.198, *p* = 0.0044), diet (*F* = 27.326, *p* < 0.001), and a significant interaction between strain and diet (*F* = 9.481, *p* < 0.01). This suggests a nuanced interplay between genetic factors and dietary influences in shaping the observed phosphorylation patterns. In contrast, the analysis of the relative phosphorylation of dimeric complex IV (IV_2_) from BN-PAGE (Figure 5G) offered a different picture. Here, the only significant main effect emerged from diet (*F* = 47.956, *p* < 0.0001), while strain type and the interaction between both factors (strain and diet) were statistically significant. These findings suggest that the relative CcO subunit I Y304 phosphorylation is intricately influenced by both diet and strain, indicating a collaborative impact of these factors. On the other hand, dimer-specific CcO subunit I Y304 phosphorylation is predominantly determined by the composition of the diet, highlighting the selective influence of dietary factors in this particular phosphorylation event.

Consistent with our observed oxygen consumption rate (OCR) patterns, we identified a notable decrease in ATP levels specifically within the CDr strain on HSD with a 50% decrease relative to CDr RD, and a 30% reduction of ATP content in both CDs RD and CDs HSD strains. The heightened phosphorylation levels in this CDr HSD were found to be correlated with a significant reduction in total liver ATP content (Figure 6C). Two-way ANOVA analysis revealed non-significant main effect of strain type but a significance main effect of dietary composition (*F* = 17.255, *p* < 0.01). Furthermore, the interaction between diet and strain was found to significantly effects ATP changes in liver tissue (*F* = 11.891, *p* < 0.01).

## 4. Discussion

In our previous research on pancreatic islets, we established a notable reduction of CcO activity in islets of the hyperglycemic-CDs that significantly correlated with β-cell dysfunction and the onset of diabetes [5,6,8]. Building upon this, we expanded our research to evaluate the influence of high-sucrose diets on mitochondrial function in the liver, using the Cohen diabetic rat model. We identified fatty liver as a complication of diabetes in hyperglycemic CDs rats in an earlier study, yet the underlying mechanism and the role of mitochondria in this phenomenon were not explored [7]. Nonalcoholic fatty liver disease is acknowledged as a prevalent complication of diabetes, elevating the likelihood of developing cirrhosis and hepatocellular carcinoma [26]. However, the underlying mechanism and optimal treatment remain to be fully elucidated.

To our knowledge, only one study has analyzed CcO function in the liver in rodent diabetes models. This study, conducted in streptozotocin treated mice and db/db mice representing type 1 and type 2 diabetes models, respectively, reported increased CcO-dependent respiration exclusively in type 1 diabetic mice fed a high-fat diet [27]. This finding may be attributed to the uncoupling of the mitochondrial membrane potential mediated by elevated levels of free fatty acids, which would stimulate mitochondrial respiration. However, the authors utilized the electron catalyst TMPD for their measurements, rendering the CcO kinetics non-physiological [28], making interpretation of the data difficult. In another proteomic study, mice were subjected to a combined high-fructose and high-fat diet to encourage diet-induced non-alcoholic fatty liver disease [29]. The authors reported mitochondrial dysregulation, characterized by a decrease in complexes I and III, yet an increase in CcO at the protein level. However, enzyme activities were not assessed. In contrast, our study demonstrates a substantial reduction in overall enzymatic activity of CcO in rats subjected to a high sucrose diet. Importantly, even under a regular diet, CDs rats exhibited a significant reduction in CcO activity. This suggests that a certain genetic and/or epigenetic makeup may predispose individuals to an inherently impaired mitochondrial respiration phenotype in the liver. Consistent with reduced CcO activity we observed reduced ATP levels in the resistant strain when on HSD and in the sensitive strain on both HSD and RD. Interestingly, the sensitive strain already showed reduced hepatic ATP levels even on a normal diet. Also noteworthy is the fact that there is mounting evidence that ATP deficiency is involved in the development of liver insulin resistance, glucose and lipid metabolism disorders, and even diabetes in cell and animal models as well as human diabetic patients [30,31,32]. Consequently, augmenting hepatic ATP synthesis has been proposed as a therapeutic strategy for the treatment of metabolic disorders including diabetes [33] and our finding of Y304 phosphorylation may provide a new mechanistic target for such an approach. Taken together, our data imply that excessive sucrose consumption leads to mitochondrial respiratory dysfunction and that factors beyond dietary intake, such as genetic predisposition, significantly contribute to the heightened susceptibility of (pre-)diabetic metabolic disorders observed in the liver. We further predict that mitochondrial reactive oxygen species (ROS) are not a major player in the liver pathology seen in this model because ROS are generated exponentially at high mitochondrial membrane potentials exceeding 140 mV (discussed in [16]). Because CcO Y304 phosphorylation leads to CcO inhibition and a reduction in the mitochondrial membrane potential [19], ROS levels should not be increased, which was also suggested by indistinguishable lipid peroxidation levels (assessed by 4-hydroxy 2-nonenal detection).

Our findings from liver tissue indicate that the HSD leads to metabolic dysfunction in the liver even in the CDr rats, that do not exhibit conventional signs or markers of diabetes after being subjected to a HSD. While the effects observed in the CDr rats were not as pronounced as those observed in the CDs group, they still showed significant deviations compared to CDr rats on a RD. The notion of mitochondrial dysfunction as a key determinant of diabetes has been previously proposed [34]. Over the years, the concept that there is a threshold of mitochondrial bioenergetic function needed for disease influenced by both genetic and environmental factors has gained wider acceptance. When this threshold is surpassed, it leads to the development of pathological diabetic phenotypes [35,36]. We propose that the mitochondrial defects seen in the CDs animals subjected to a HSD are below this mitochondrial threshold resulting in the manifestation of an overt diabetic phenotype [6].

It has been shown that mice subjected to a high-sucrose diet display signs of inflammation in their adipose tissue and liver, marked by elevated levels of pro-inflammatory cytokine TNFα [37], a cytokine associated with adipocyte enlargement, fat accumulation, insulin resistance, and elevated blood sugar levels. Previously, we discovered that TNFα significantly decreased CcO activity in mouse liver tissue through downstream phosphorylation of Y304 residue of CcO catalytic subunit I, resulting in a sharp decline in mitochondrial membrane potential and a reduction in cellular ATP levels [19]. In the current study, the utilization of BN-PAGE provided a deeper insight into the dynamic alterations occurring within protein complexes and their enzymatic activities. CcO-Y304 phosphorylation was found to be specific for dimeric CcO and is associated with CIV_2_ enzymatic inhibition. This finding supports our earlier conclusions, which indicated sigmoidal CcO kinetics for pY304-phosphorylated CcO with Hill coefficients approaching 4, which can only be explained by the presence of CcO dimers as we have discussed [18]. Phosphorylation is one of several PTMs that alters protein conformation and function and is used by the cell to modulate enzymatic function. Phosphorylation of Y304 on CcO subunit I demonstrated here upon HSD feeding provides a molecular mechanism of mitochondrial dysfunction for the first time and offers insights into the response of liver tissue during hyperglycemia and in the (pre-)diabetic liver. Phosphorylation of CcO subunit I Y304 may mediate mitochondrial dysfunction in response to elevated blood sugar levels, linking metabolic changes to mitochondrial impairments, and it could potentially serve as a marker for the detection of early diabetic liver dysfunction.

The efficiency, activity, and flux within the electron transport chain is regulated at several levels. In addition to PTMs, the abundance, stability, and compositions of supercomplexes can play a role for the liver to modulate energy production during metabolic stress such as HSD. Within the HSD groups, a notable shift in the activity of Complex I was observed, especially in higher order supercomplexes (I+III_2_+IV_n_). This observed alteration in complex I activity may be attributed to a compensatory response arising from the inhibitory phosphorylation of CIV_2_, and it suggests a dynamic interplay between different components of the mitochondrial electron transport chain. The finding that pY304 phosphorylation is primarily found in the inactive CcO dimer suggest that similar studies should be carried out to analyze changes in SCs assembly more broadly through PTMs, including other tissues that are heavily implicated in diabetes.

## 5. Conclusions

**Figure 7:**
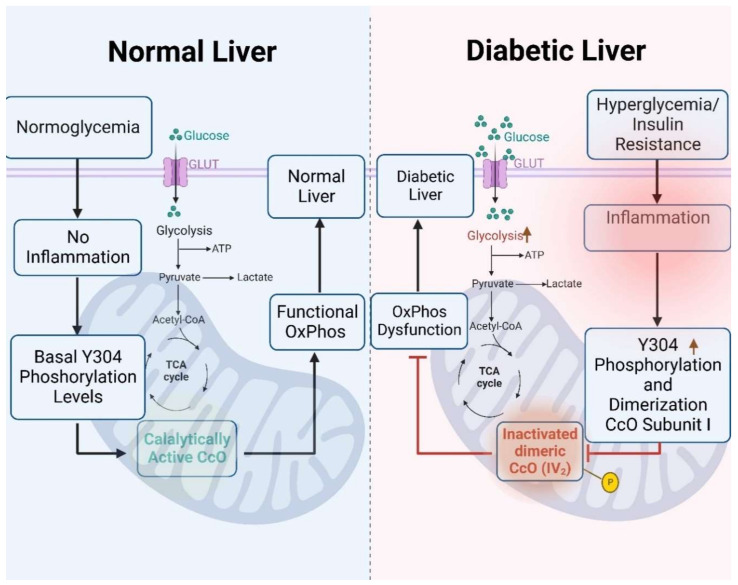
Model of mitochondrial dysfunction associated with tyrosine 304 phosphorylation of subunit I of dimeric complex IV during hyperglycemia in rat liver tissue.

In this study, we have uncovered the presence of a specific inhibitory phosphorylation on tyrosine 304 of catalytic subunit I in dimeric CcO. This phosphorylation causes a decrease in enzymatic activity and respiratory dysfunction in rat liver tissue under hyperglycemic conditions. The mitochondrial oxidative phosphorylation process generates most of the cellular energy and free radicals in mammalian tissues. Both factors play a critical role in numerous human diseases that could be affected by reversible phosphorylation events of the oxidative phosphorylation complexes. We propose a model in which this Y304 phosphorylation drives CcO dimer formation as a downstream effect of inflammatory signaling that is associated with diabetes, leading to a profound reduction of CcO enzymatic activity in (pre-)diabetic liver tissue (Figure 7).

## Figures and Tables

**Table 1:** Characterization of blood glucose levels 2 h post OGTT and body weight.

Group	n	2 h Post-OGTT (mmol/L)	Body Weight (g)
CDr RD	5	7.13 ± 1.02	328.3 ± 7.8
CDr HSD	5	7.44 ± 0.18	292.3 ± 5.0
CDs RD	5	7.69 ± 0.1	306.5 ± 5.4
CDs HSD	5	16.3 ± 1.6 *	301.0 ± 12.9

* Only CDs HSD rats presented with a diabetic phenotype based on OGTT.

**Table 2:** List of antibodies used in this study.

Antibody	Dilution	Company	Product ID
Beta-actin	1:2000	Proteintech	60008-1-Ig
VDAC1/2	1:5000	Proteintech	10866-1-AP
NDUFB6	1:5000	Abcam	ab110244
Core I	1:3000	Abcam	ab110252
MT-CO1 (CcO I)	1:5000	Invitrogen	PA5-68016
Custom-made pY304 CcO I	1:3000	Abgent	RB 96601
Anti-rabbit IgG HRP-linked	1:5000	Cell Signaling	7074S
Anti-mouse IgG HRP-linked	1:5000	Cell Signaling	7076S

## Data Availability

The data presented in this study are available in the article.

## Supplementary Materials

The following supporting information can be downloaded at: https://www.mdpi.com/article/10.3390/antiox13010019/s1, Figure S1: Complex I-related in-gel activity is unaltered in the I+III_2_ supercomplex; Figure S2: Decrease in CcO subunit I content in HSD strains; Table S1: Summary of Two-Way ANOVA results of Figure 3.
