# Supplementary material for: High Sucrose Diet-Induced Subunit I Tyrosine 304 Phosphorylation of Cytochrome c Oxidase Leads to Liver Mitochondrial Respiratory Dysfunction in the Cohen Diabetic Rat Model
Source: Antioxidants (Basel). 2023 Dec 21;13(1):19. doi: 10.3390/antiox13010019 (PMC10812566; doi:10.3390/antiox13010019)
Supplement: Supplementary file 1 [file antioxidants-13-00019-s001.zip › antioxidants-2738323-supplementary.pdf]

Supplementary Figures

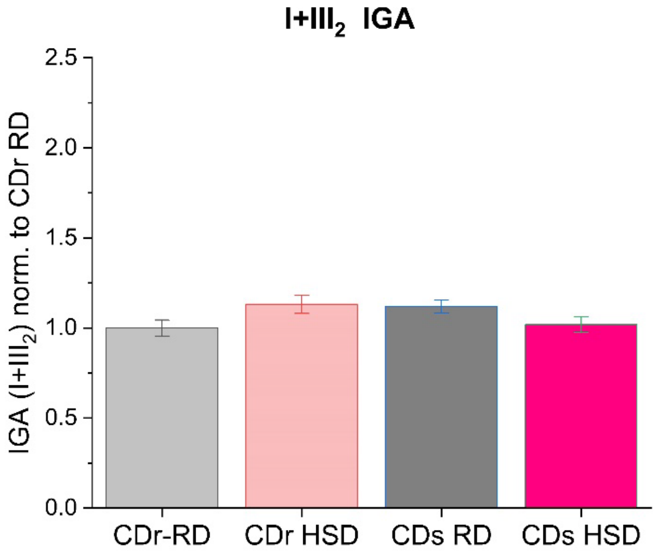

Figure S1. Complex I-related in-gel activity is unaltered in the I+III2 supercomplex. Data are reported as % of CDr RD set to 100% and derived from three independent experiments using mitochondria from three distinct Cohen diabetes-resistant (CDr) and Cohen diabetes-sensitive (CDs) rat livers. RD, regular diet; HSD, high sucrose low copper diet.

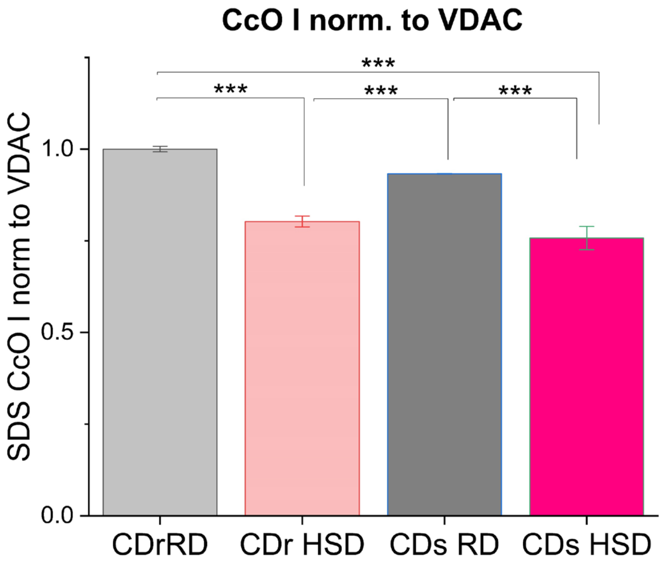

Figure S2. Decrease in CcO subunit I content in HSD strains. Quantitation of CcO subunit I normalized to total VDAC. Data are reported as % of CDr RD set to 100% and derived from three independent experiments using mitochondria from three distinct Cohen diabetes-resistant (CDr) and Cohen diabetes-sensitive (CDs) rat livers. RD, regular diet; HSD, high sucrose low copper diet; ***, $p < 0{,}001$.

Supplementary Tables

Table S1. Summary of Two-Way ANOVA results of Figure 3.

	IGA Band	Strain Effect	Diet Effect	Interaction Effect
Complex IV IGA	IV	ns.	F= 44.941<br>p < 0.001	F= 10.267<br>p= 0.003
	IV_2	ns.	F= 26.597<br>p < 0.001	F= 10.521<br>p= 0.001
	III_2+IV	ns.	F= 18.438<br>P < 0.001	F= 7.365<br>p= 0.001
Complex I IGA	I	ns.	F= 11.736<br>p < 0.003	ns.
	I+III_2	ns.	ns.	F= 7.031<br>p=0.02
	I+III_2+IV	ns.	ns.	F= 21.21<br>p < 0.001
	I+III_2+IV_n	ns.	F= 16.640<br>p < 0.001	ns.

ns: not significant
